# Tumor architecture exerts no bias on nuclear grading in breast cancer diagnosis

**DOI:** 10.1007/s00428-012-1304-1

**Published:** 2012-08-31

**Authors:** Braulio Mora, Dario Bombari, Stephan C. Schaefer, Marcus Schmidt, Jean-Francois Delaloye, Fred Mast, Hans-Anton Lehr

**Affiliations:** 1 Institute of Pathology, CHUV, Lausanne, Switzerland; 2 Department of Psychology, University of Bern, Bern, Switzerland; 3 Institute of Pathology, Inselspital, Bern, Switzerland; 4 Department of Gynecology and Obstetrics, University of Mainz, Mainz, Germany; 5 Department of Gynecology and Obstetrics, CHUV, Lausanne, Switzerland; 6 Insitut universitaire de pathologie, Centre Hospitalier Universitaire Vaudois, Rue du Bugnon 25, 1011 Lausanne, Switzerland

**Keywords:** Confirmation bias, Cancer grading, Nuclear pleomorphism, Architecture, Cognitive psychology

## Abstract

We recently reported that nuclear grading in prostate cancer is subject to a strong confirmation bias induced by the tumor architecture. We now wondered whether a similar bias governs nuclear grading in breast carcinoma. An unannounced test was performed at a pathology conference. Pathologists were asked to grade nuclei in a PowerPoint presentation. Circular high power fields of 27 invasive ductal carcinomas were shown, superimposed over low power background images of either tubule-rich or tubule-poor carcinomas. We found (a) that diagnostic reproducibility of nuclear grades was poor to moderate (weighed kappa values between 0.07 and 0.54, 27 cases, 44 graders), but (b) that nuclear grades were not affected by the tumor architecture. We speculate that the categorized grading in breast cancer, separating tubule formation, nuclear pleomorphism, and mitotic figure counts in a combined three tier score, prevents the bias that architecture exerts on nuclear grades in less well-controlled situations.

## Introduction

Despite major advances in our understanding of breast cancer biology and the advent of novel diagnostic tools to predict survival and treatment response of breast cancer patients (i.e., MIB-1, IGkG, genetic tests, etc.) [1–3], time-honored tumor grading has been confirmed as one of the most reliable prognostic and predictive markers [4]. Grading of breast carcinomas is based on three histomorphological criteria: degree of tubular differentiation, nuclear pleomorphism, and mitotic figure count [5, 6]. Recently, we found that nuclear grading in prostate cancer is subject to a strong confirmational bias induced by the tumor architecture: pathologists unconsciously assigned grades for nuclear pleomorphism that matched the Gleason grade—irrespective of nuclear morphology [7, 8]. This phenomenon is of little clinical consequence in prostate cancer, where prognosis is predicted by tumor architecture and not by nuclear grade [7]. However, we wondered whether the same heuristic phenomenon could be operative in the grading of breast cancer, potentially leading to a clinically relevant loss of prognostic/predictive information.

## Methods

**Fig. 1:**
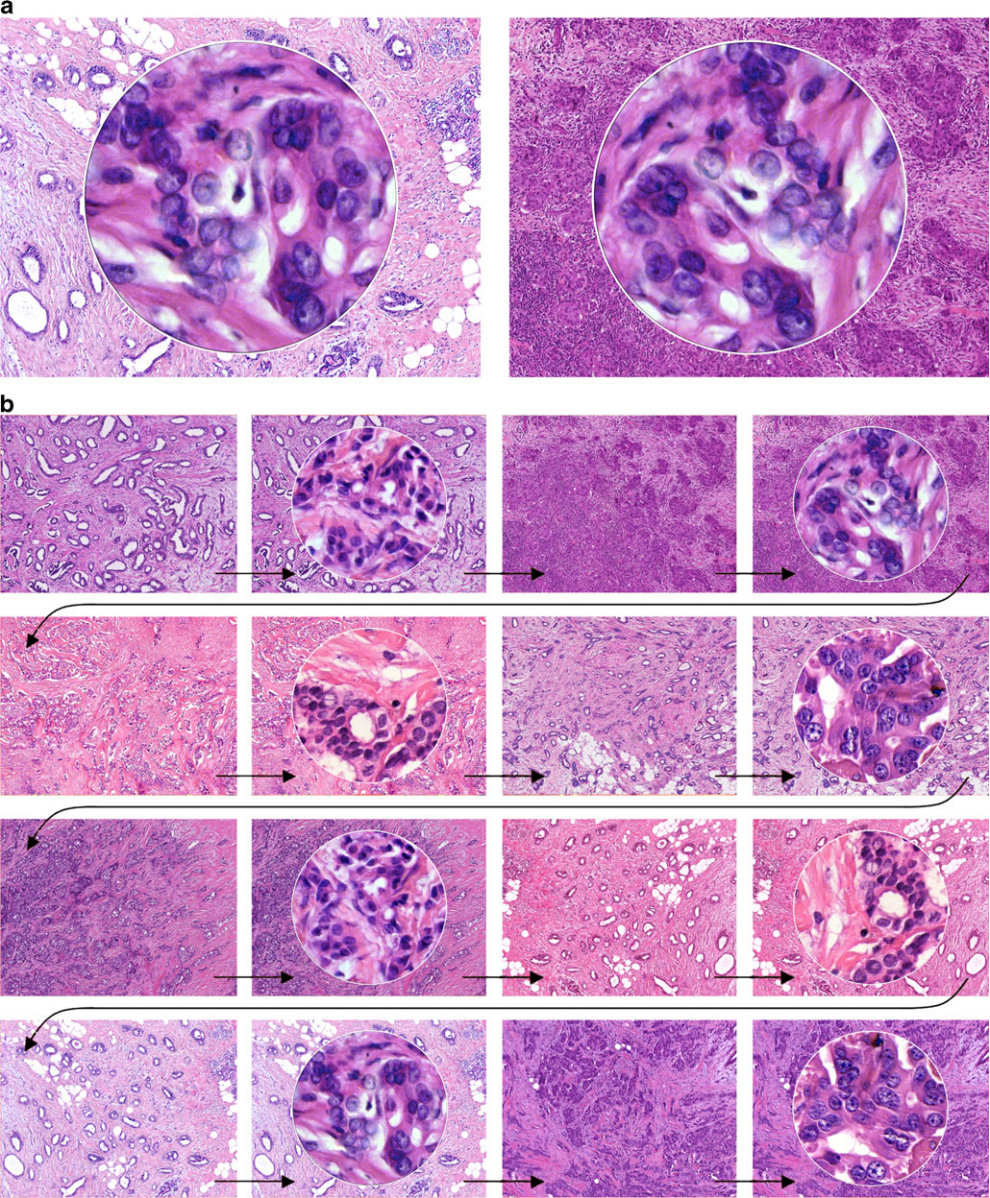
PowerPoint presentation showing high power fields superimposed over different architectural backgrounds. Using a beamer, HPFs of 27 invasive ductal carcinomas were projected in the plenary lecture hall of a pathology conference. Unknown to the participants, each high power filed was shown twice, superimposed once over a well-differentiated, tubule-rich background image and once over a poorly differentiated, tubule-poor background image (**a**). In order to avoid recognition, high power fields were flipped and rotated within their circular inset (**a**). The presentation was automatically timed to show each HPF for 7 s. Between HPFs, the background image of the next case was shown for 1 s (**b**). Pathologists were asked to assign nuclear grades for each HPF and note their grade on a form that they found on their seats. The present figure was specifically created for this publication to demonstrate the principle, but using only a limited set of only four high power fields, each shown twice, superimposed over the distinct (tubule-rich or tubule-poor) background images. In the original presentation, a total of *n* = 27 cases were randomly presented

In analogy to the prostate cancer experiments [8], we prepared a PowerPoint presentation, where circular images of high power fields (HPF, *Ø*0.62 mm) of 27 invasive ductal carcinomas were superimposed over low power background images with the tumor architecture. The photographs were taken from a historical collection of cases from 1993 to 1995 at the University of Mainz [2, 4], for which specific patient consent had been obtained. No patient identifiers were known. In a random order, each HPF image was shown twice, once superimposed over a background image of a well-differentiated, tubule-rich carcinoma and once superimposed over an image of a poorly differentiated, tubule-poor carcinoma (Fig.1a). In order to avoid recognition of the HPFs after the first presentation, the circular images belonging to the same case were rotated and flipped using Photoshop (Adobe, CS3, St. Jose, CA). With permission from the organizers of the joint annual meeting of the Austrian and the Swiss Societies of Pathology in Feldkirch in November 2010, this presentation was shown to the audience of a plenary session. Each HPF was displayed for 7 s, followed by a 1-s transition in which the background image of the next case was shown (Fig.1b). The 7 s was chosen based on previous experience with a similar experimental setup, where nuclei were displayed on a computer screen for 8 s, and where the pathologists spoke out loud the nuclear grade at an average of 4–5 s into the 8-s interval. The presentation of the 60 HPF hence lasted exactly for 8 min. The experiment had not been announced (it “spontaneously” replaced a talk that was printed in the program book). In a brief introduction, pathologists were asked to grade nuclear pleomorphism of the projected HPFs on a five-tier scale (1, 1–2, 2, 2–3, and 3) and note the grades on a form that they found on their seats. No information was given concerning the true aim of the experiment. In particular, no mention was made of the background images. It was hence left to the “imagination” of the participants to assume that the background images belonged to the high power fields. This was the exact same experimental situation that we had used before in the prostate cancer experiments in which we had found a strong architectural bias on nuclear grading [8]. The only additional information that was asked from the participants was the number of years in practice. Statistical analyses included weighed kappa analyses and paired *t* tests (www.wessa.net).

## Results

Fifty-eight of the conference attendees (70–80) participated in the experiment. Data from 14 participants were discarded since they did not rate all HPFs. Thirty-six of the remaining 44 raters indicated the number of years of experience. The reliability of nuclear grade assignment ranged from moderate to good for about half of the HPFs (*n* = 14, weighed kappa values 0.25–0.54) and poor for the other half of the HPFs (*n* = 13, weighed kappa values 0.07–0.25). Grading consistency was not affected by the years of professional experience (data not shown).

Average nuclear grades assigned to the HPFs superimposed over tubule-rich backgrounds (2.10 ± 0.63) were comparable to average grades assigned to the same HPFs superimposed over tubule-poor backgrounds (2.15 ± 0.62), suggesting that tumor architecture did not influence nuclear grade assignment. Also, the difference of the tubular differentiation of the background images did not influence nuclear grades (1.92 ± 0.63 for 16 HPFs presented before pure tubular backgrounds vs. 2.00 ± 0.67 for the same HPFs presented before solid backgrounds, n.s.).

**Fig. 2:**
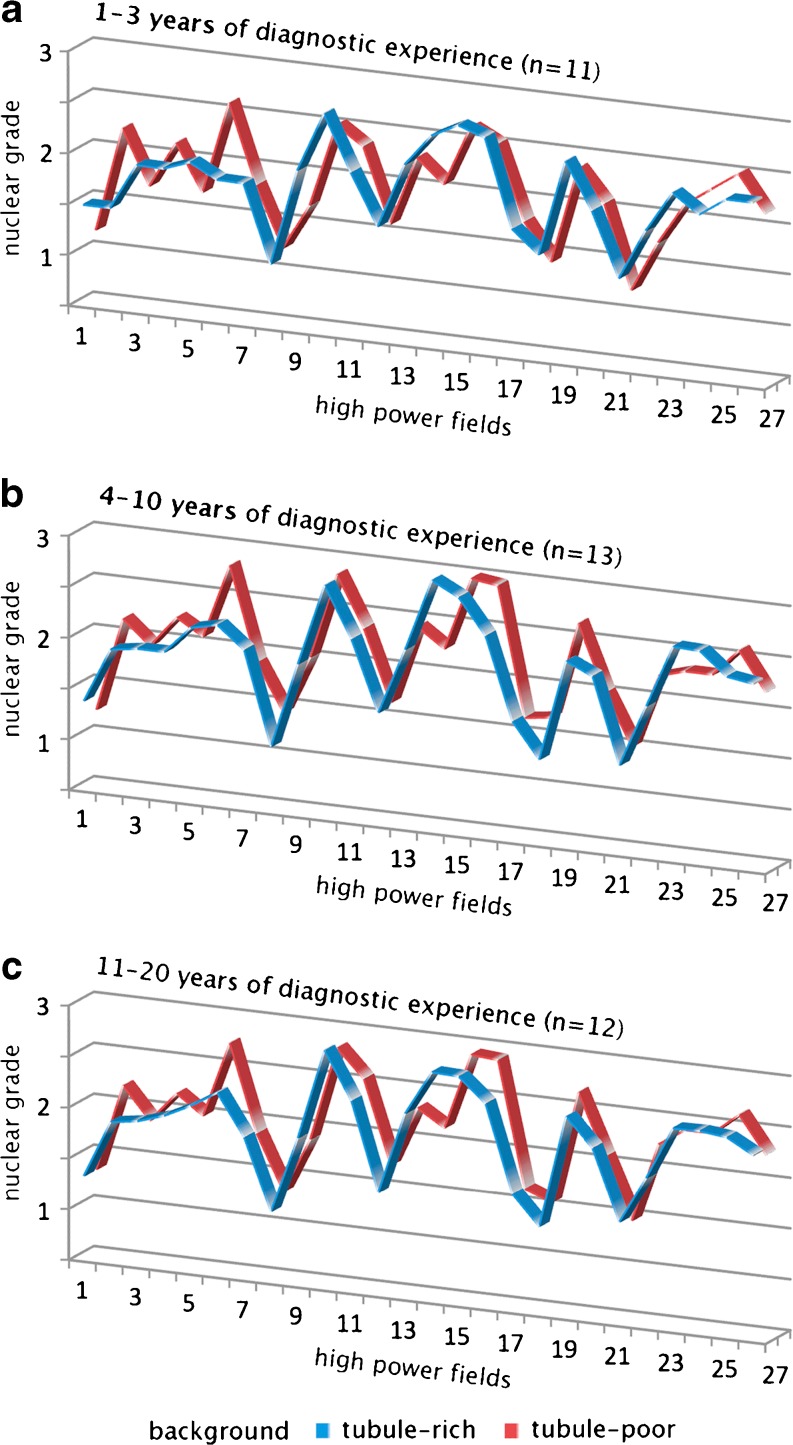
Nuclear grades assigned to 27 pairs of high power fields superimposed over tubule-rich (*blue*) or tubule-poor (*red*) backgrounds. The data are shown for three subgroups of pathologists with various years of experience. **a** 1–3 years of experience, *n* = 11; **b** 4–10 years of experience, *n* = 13; **c** 11–20 years of experience, *n* = 12. Note that for most high power fields, pathologists assigned similar nuclear grades, largely unaffected by the architecture of the background images

We also found that the professional experience of the participants had no influence on the results: neither did we see any effect of tubule formation on nuclear grades in *n* = 11 beginners (1–3 years of professional experience; 2.03 ± 0.42 vs. 1.99 ± 0.44), in *n* = 13 more experienced pathologists (4–10 years of experience; 2.13 ± 0.48 vs. 2.16 ± 0.47), nor in *n* = 12 “old-timers” with more than 10 years of experience (2.11 ± 0.46 vs. 2.15 ± 0.47). These results are shown in a graphic form in Fig. 2.

## Discussion

The principal finding of this experiment is that grading of nuclear pleomorphism in invasive ductal breast carcinoma is not affected by the tumor architecture. This finding is unexpected, as it does not confirm our prior observations in prostate carcinomas [7, 8]. In these previous studies, we found that nuclear grade assignment was unconsciously affected by a powerful confirmation bias induced by the tumor architecture. In Bombari's study [8], each of 20 pathologists was assigned lower pleomorphism grades to the nuclei of high power fields when these had been superimposed over a background of a tubule-rich carcinoma (Gleason pattern 3) and significanly higher grades when the same high power fields were shown a little later superimposed over an image of a cribriform or solid carcinoma (Gleason patterns 4 or 5). Tracking pathologists' eye fixations further showed that depending on the tumor architecture of the background image, pathologists looked at different nuclei within the high power fields: they fixated on smaller nuclei with evenly distributed, pale chromatin when the background showed a Gleason 3 pattern and on larger, hyperchromatic nuclei when the background was a Geason 4 or 5 pattern [8]. However, this selection of nuclei that “matched the expectation” accounted for only about one tenth of the total grade gravitation, suggesting that nuclear selection represents an unconscious strategy of our minds to vindicate the confirmation bias induced by the tumor architecture [8].

In the present study, we found that this powerful confirmation bias is not operative in breast cancer grading, even though the participants had exactly the same chance to explore tumor architecture and nuclear pleomorphism on the projected tumor images as they had in the previous experiments on prostate cancers [8]. In analogy to well-established modes of avoiding optical illusions by the adherence to strict guidelines and rules in aviation [9], we assume that the rigid grading system of breast cancer that was initially established by Bloom and Richardson [5] and later modified by Elston and Ellis [6] effectively prevents cross-category biases. By experience, pathologists know that in breast cancer, architecture and nuclear grade do not necessarily go along hand in hand. For instance, lobular carcinomas show no tubular differentiation (grade 3), but usually little nuclear pleomorphism (grade 1). In contrast, every pathologist has seen carcinomas with “ugly” nuclei (grade 3), but with more or less well-formed tubular architecture (grade 1). In breast cancer diagnosis, it hence poses no “conflict” for our minds to see small, round, and pale nuclei and correctly assign them a grade 1 even though the low power image in the background displays an architecturally undifferentiated (grade 3) carcinoma without any formation of tubules. In this context, it should be noticed that the pathologists who participated in the study all practice in Austria and in Switzerland, where the adherence to the three tier Bloom and Richardson grading system is generally very high. Also, it may be of interest to note that most pathologists in these two countries practice in a rather “generalist” approach, with only few departments being organized in subspeciality services, and that the session, in which the experiment was performed, had no particular breast focus. This suggests that the results of our study can be applied to most diagnostic pathologists.

In this experiment, we have focussed on the degree of tubule formation as leading architectural pattern. Another histological feature that may have potentially biased the partcipants is the relative amount of stroma between tumor cells. Pathologists tend to be quite vigilant to morphological cues that contribute, sometimes quite unconsciously, to the interpretation of the complex image on the H&E-stained slide. These cues are not always easily quantifiable, such as the percent of tubule formation or the number of mitotic figures, but are often quite subjective in nature, such as the presence and the extent of necrosis, the type and amount of peritumoral stroma, or the peritumoral lymphocytic response, to name only a few of the more obvious features. In a less well-controlled situation, as we have identified recently in prostate cancer grading [7, 8], such additional morphological features will likely affect overall cancer grading. However, the findings of the present study, where tubule formation did not affect nuclear grading (Fig. 2), suggest that in breast cancer grading, where the well-standardized and widely applied Bloom and Richardson criteria separate architectural features from nuclear features and from mitotic figure counts, stroma will likely not affect nuclear grading.

The second observation that we made in the present study is that grade assignment to nuclei in high power fields is surprisingly inaccurate. We used weighed kappa analysis to quantify the consistency of grade assignment to the 27 pairs of high power fields. The interobserver reliability was poor in about half of the cases (weighed kappa values below 0.25) and moderate to good in the other half of the cases (kappa values between 0.25 and 0.54). We acknowledge that the experimental setup used in this study had a different primary aim. Also, grade assignment on a single projected high power field certainly does not reflect the situation that pathologists encounter during routine cancer grading, where they scan thousands of nuclei during nuclear grade assignment and not only those in one high power field. Nevertheless, our findings correlated rather well with kappa scores for interobserver reliability of nuclear pleomorphism in prior reports (range 0.3 to 0.4) that have underscored the limitations of this particular component of the grading system in breast cancer [10–12].

To conclude, we found that a cognitive bias induced by the tumor architecture does not govern nuclear grading in breast cancer diagnosis. Even though more research is needed to determine the exact conditions under which this confirmation bias unfolds, the present observation is relevant in that it suggests that adherence to strict guidelines, notably categorized grading, has the capacity to effectively counteract errors due to inconscious mental shortcuts in histological cancer grading.
